# GaAs-chip-based mid-infrared supercontinuum generation

**DOI:** 10.1038/s41377-023-01299-9

**Published:** 2023-10-18

**Authors:** Geoffroy Granger, Myriam Bailly, Hugo Delahaye, Cristian Jimenez, Idris Tiliouine, Yann Leventoux, Jean-Christophe Orlianges, Vincent Couderc, Bruno Gérard, Rezki Becheker, Said Idlahcen, Thomas Godin, Ammar Hideur, Arnaud Grisard, Eric Lallier, Sébastien Février

**Affiliations:** 1 Université de Limoges XLIM UMR CNRS 7252, 123 Av. A. Thomas, 87060 Limoges, France; 2 Thales Research & Technology, 1 Av. Augustin Fresnel, 91767 Palaiseau Cedex, France; 3 III-V Lab, 1 Av. Augustin Fresnel, 91767 Palaiseau Cedex, France; 4 CORIA (UMR 6614), CNRS-INSA Rouen-Université de Rouen Normandie, Normandie Université, Saint-Etienne du Rouvray, France

**Keywords:** Ultrafast lasers, Supercontinuum generation

## Abstract

The mid-infrared spectral region opens up new possibilities for applications such as molecular spectroscopy with high spatial and frequency resolution. For example, the mid-infrared light provided by synchrotron sources has helped for early diagnosis of several pathologies. However, alternative light sources at the table-top scale would enable better access to these state-of-the-art characterizations, eventually speeding up research in biology and medicine. Mid-infrared supercontinuum generation in highly nonlinear waveguides pumped by compact fiber lasers represents an appealing alternative to synchrotrons. Here, we introduce orientation-patterned gallium arsenide waveguides as a new versatile platform for mid-infrared supercontinuum generation. Waveguides and fiber-based pump lasers are optimized in tandem to allow for the group velocities of the signal and the idler waves to match near the degeneracy point. This configuration exacerbates supercontinuum generation from 4 to 9 µm when waveguides are pumped at 2750 nm with few-nanojoule energy pulses. The brightness of the novel mid-infrared source exceeds that of the third-generation synchrotron source by a factor of 20. We also show that the nonlinear dynamics is strongly influenced by the choice of waveguide and laser parameters, thus offering an additional degree of freedom in tailoring the spectral profile of the generated light. Such an approach then opens new paths for high-brightness mid-infrared laser sources development for high-resolution spectroscopy and imaging. Furthermore, thanks to the excellent mechanical and thermal properties of the waveguide material, further power scaling seems feasible, allowing for the generation of watt-level ultra-broad frequency combs in the mid-infrared.

## Abstract (web summary)

Orientation-patterned GaAs waveguide and mid-infrared picosecond pump laser are used in tandem to produce octave-spanning quadratic supercontinuum.

## Graphical abstract

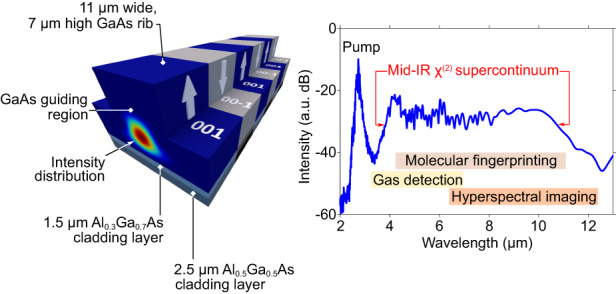

## Introduction

High repetition rate (>MHz) sources of broadband light pulses in the middle-wave infrared (mid-IR, 3 to 25 µm) open up new possibilities for various applications such as molecular spectroscopy^1–3^, high resolution imaging^4,5^ and high-harmonic generation in condensed matter^6–8^. Synchrotron sources provide radiation in a very broad electromagnetic spectrum, from the X-rays to the infrared, with high brightness. The infrared light is used for analyzing the biochemical state and chemical content of biological tissues at micrometer scale resolution. This high precision identification and chemical imaging has helped for early diagnosis of several pathologies. However, high cost and limited availability have hindered the broad use of synchrotron-based techniques. A compact laser source, which covers the wavelength range identified relevant by initial synchrotron studies, could unleash the potential of mid-IR spectromicroscopy imaging techniques in science and technology. Although remarkable breakthroughs towards the direct generation of broadband mid-IR pulses were recently reported in quantum cascade lasers (QCL)^9,10^, their performances are not yet compatible with the high-power applications mentioned above and the spectral bandwidth of such QCL combs is still very narrow. Frequency down-conversion in nonlinear materials therefore remains as the preferred choice for mid-IR generation. In particular, much attention was paid in the last decade to supercontinuum generation in mid-IR transparent glass optical fibers with χ^(3)^ nonlinearity. By leveraging the fluoride fiber technology, robust and versatile high-repetition rate (multi-MHz) sources emitting up to ~5 µm have become commercially available and are nowadays exploited in high-resolution spectromicroscopy^5^ and optical coherence tomography^11^. Furthermore, chalcogenide fibers have been used to extend the spectral coverage into the mid-IR. Two octave spanning supercontinuum generation with brightness exceeding that of the synchrotron source was obtained in chalcogenide fibers pumped by ultrafast laser sources^12,13^. However, the large nonlinearity and poor thermal properties of the mid-IR fibers limit the power scalability of the sources. Another successful approach for the generation of broadband light in the mid-IR relies on the exploitation of the large nonlinearity of binary alloys of either Column IV semiconductors (Si, Ge) or III-V semiconductors (e.g. Ga, As), which offer wideband transmission across the mid-IR as well as high optical nonlinearity. Large bandwidth supercontinuums were demonstrated recently in various designs of Si-Ge^14,15^ and AlGaAs^16^ waveguides and, in a lower extent in silicon nitride waveguides^17^, owing to their large χ^(3)^ nonlinearity. However, the generation of broadband mid-IR supercontinuum from such platforms requires the use of ultrashort light pulses centered at wavelengths >4 µm which calls for complex laser systems to be produced^14,15^. On another hand, orientation-patterned gallium arsenide (OP-GaAs) has proven to be an efficient nonlinear material combining a strong nonlinear response^18,19^ with a large transparency window, and then suitable for the generation of broadly tunable mid-IR radiation^20^. In particular, the combination of the quasi-phase-matched (QPM) geometry with the degenerate pumping approach (i.e. pumping near half the zero-group velocity dispersion (GVD) wavelength of the material) is very appealing for the generation and amplification of broad bandwidths of radiation^21^. This concept, first demonstrated with bulk OP-GaAs, enabled broadband optical parametric generation (OPG) spanning from 4.5 to 10.7 µm at -20 dB of the peak intensity when pumped at 3.3 µm by a 2 kHz optical parametric amplifier^22^. Despite the excellent mechanical and thermal properties of the material, the approaches based on bulk OP-GaAs offer a limited usability due to the high threshold and low efficiency of the OPG process, which calls for cumbersome powerful laser pump sources. Conversely, the OP-GaAs waveguide architecture overcomes these limitations and constitutes an ideal candidate for high efficiency down-conversion to the mid-IR^23^ using highly integrated ultrafast fiber-based pump sources. The tight confinement of light in GaAs waveguides indeed allows for an increased power spatial density over centimeter long interaction distances, thereby relaxing the constraints on the energy and therefore on the nature of the pump source. We recently designed and fabricated OP-GaAs/AlGaAs waveguides with reduced losses of 3 dB.cm^−1^ at 8 µm^24^ and demonstrated OPG from low-energy fluoride fiber laser pumps^25^. In this communication, we combine OP-GaAs/AlGaAs waveguides with fiber laser pumping to exploit an anomaly in the phase-matching curve of optical parametric processes^26^ and generate an octave-spanning mid-IR supercontinuum with a power spectral density (PSD) exceeding that of the synchrotron radiation source by one order of magnitude within a table-top setup.

## Results

### OP-GaAs waveguide

**Fig. 1:**
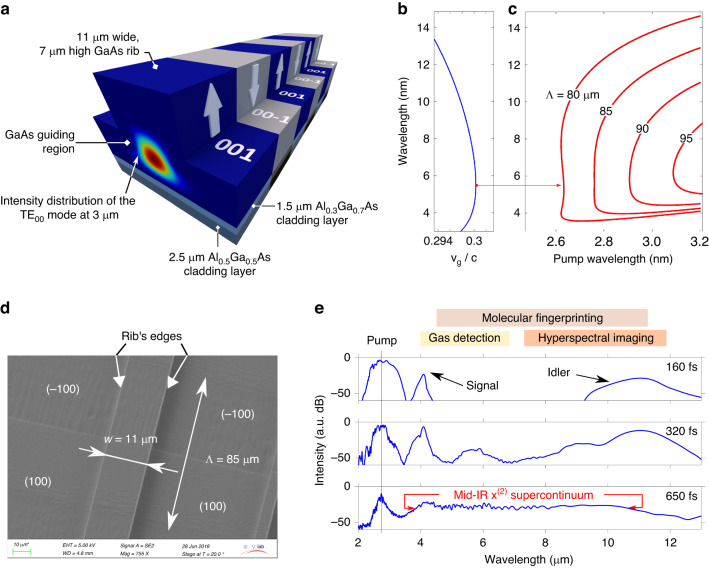
**Numerical study of group-velocity matching mediated supercontinuum generation in orientation-patterned gallium arsenide waveguide. a** Schematic depicting the OP-GaAs/AlGaAs waveguide used in our experiments. Vertical arrows labeled [001] and [00-1] refer to the crystallographic orientation reversed every half QPM period Λ. **b** Group velocity *v*_g_ of the TE_00_ mode guided in the selected waveguide. The zero-dispersion point, represented as a red dot, is located at 5.5 µm. **c** Phase-matching curves calculated for various QPM periods Λ as a function of pump wavelength in the selected waveguide with *w* = 11 µm. QPM periods of 80–85 µm are optimal choices for flat supercontinuum generation when pump wavelength lies around half the zero-dispersion wavelength, in between 2.6 and 2.8 µm. **d** Scanning electron microscope top view of a waveguide showing the two crystallographic domain orientations [001] and [00-1] and the dimensions. The two arrows show the edges of the rib. **e** Numerical study of the influence of the pump pulse duration on the OPG process when 12 kW peak power pulses at 2.76 µm are launched into the waveguide with *w* = 11 µm and Λ = 85 µm. Three values of pulse duration were studied: 160 fs, 320 fs and 650 fs

The principle of group-velocity matching mediated supercontinuum generation in orientation-patterned gallium arsenide waveguides is depicted in Fig. 1.

The intense narrow-band laser pump with frequency ${\omega }_{p}$ described in the next paragraph excites the fundamental TE_00_ mode of the waveguide with propagation constant $\beta \left({\omega }_{p}\right)={\beta }_{p}$ and is frequency converted to the signal and idler waves with lower frequencies ${\omega }_{s}$ and ${\omega }_{i}$ and propagation constants $\beta \left({\omega }_{s}\right)={\beta }_{s}$ and $\beta \left({\omega }_{i}\right)={\beta }_{i}$. The pump and generated waves satisfy both the law of conservation of energy ${\omega }_{s}+{\omega }_{i}={\omega }_{p}$ and the law of conservation of momentum. The efficiency of the parametric process is increased through QPM where the phase mismatch $\Delta \beta ={\beta }_{p}-{\beta }_{s}-{\beta }_{i}$ is compensated for at some frequencies by the periodic reversal of the GaAs crystallographic orientation with period Λ. Broadband optical parametric generation is obtained by first optimizing the pump frequency for the group velocity of the signal and idler waves to match in the vicinity of the degeneracy point where ${\omega }_{s}={\omega }_{i}={\omega }_{p}/2$. This condition is fulfilled when the signal and idler frequencies match with the zero-dispersion wavelength ${\omega }_{0}$, where the group velocity slowly varies with respect to wavelength. Then, the QPM period Λ is optimized to cancel the residual phase-mismatch (${\beta }_{p}-{\beta }_{s}-{\beta }_{i}-\frac{2\pi }{\Lambda }=0$) and to ensure efficient OPG in the regime of group velocity matching. As shown in Fig. 1a, we have studied thick OP-GaAs/AlGaAs rib waveguides^27^ fabricated according to the process derived from our experience on bulk OP-GaAs crystals^28^ (see “Materials and methods” for a detailed description of the fabrication process of the waveguides). The opto-geometrical parameters of these thick rib waveguides were selected for efficient waveguiding across the transparency window of the material. In our study, the guiding rib with width *w* varying from 7 to 12 µm is surrounded by air on three sides and by two Al_*x*_Ga_1-*x*_As layers underneath. The height of the guiding rib is 7 µm. The Al concentration in the first (resp. second) cladding layer is *x* = 0.3 (resp. 0.5), while the thicknesses of the Al-doped layers are equal to 1.5 µm and 2.5 µm. Contrary to bulk crystal, the waveguide geometry offers extra degrees of freedom in tailoring the dispersion relation β(λ). In particular, the zero-dispersion wavelength can be adjusted by varying the rib width *w*. For example, the group velocity curve computed for the TE_00_ mode of the waveguide with *w* = 11 µm is shown in Fig. 1b. The group velocity dispersion cancels at 5.5 µm, indicating that a pump wavelength of 2.75 µm is required to match the degeneracy point (${\omega }_{s}={\omega }_{i}={\omega }_{p}/2$) with the region of slowly varying group velocity. We have chosen this value for the rib width as the optimal pump wavelength can be conveniently obtained from erbium-doped fluoride fiber lasers. The law of momentum conservation applied to the propagation constant β in the waveguide geometry at the degeneracy point ($\Delta \beta ={\beta }_{p}-{2\beta }_{s,i}-\frac{2\pi }{\Lambda }=0$) then indicates that the QPM period $\Lambda$ must be chosen so that $\Lambda =\frac{1}{\frac{{n}_{p}}{{\lambda }_{p}}-\frac{2{n}_{s}}{{\lambda }_{s}}}$, where *n*_p_ and *n*_s_ are the effective indices of the TE_00_ mode at the pump and signal (or idler) wavelengths, respectively. Computation of the effective index curve of the TE_00_ mode at room temperature by the finite element method yields a value for the QPM period around 82.6 µm. This preliminary calculation is confirmed in Fig. 1c where it is shown that a QPM period of 80–85 µm in combination with a pump wavelength of 2.6–2.8 µm indeed allow for the generation of a flat spectrum from 4 to 10 µm. In our fabrication process, the QPM was obtained by a periodic reversal of the GaAs orientation from [001] to [00-1], as shown by the vertical arrows in Fig. 1a. A scanning electron microscope top view of the fabricated waveguide with two adjacent domains of opposite orientation is shown in Fig. 1d. It is worth noting the high quality of the rib’s edges, indicating a low surface roughness and therefore low propagation losses (measured to 3 dB.cm^−1^ at 8 µm). Using the nonlinear envelope equation (NEE) formalism introduced by Genty et al. for χ^(3)^ nonlinear media^29^ and by Conforti et al. for χ^(2)^ nonlinear crystals^30^, we have modeled the generation of supercontinuum in the fabricated OP-GaAs/AlGaAs waveguide with both quadratic and cubic contributions. Figure 1e shows three spectra computed for a 14.5 mm long waveguide with *w* = 11 µm and Λ = 85 µm when pump pulses with 12 kW peak power at 2.76 µm with various durations are launched into the TE_00_ mode of the waveguide. It is worth noting that short pump pulses (e.g. 160 fs and 320 fs) do not lead to supercontinuum generation due to temporal walk-off between the pump wave and the signal and idler waves induced by their large group velocity mismatch of 100 fs.mm^−1^. On the other hand, supercontinuum generation is observed for 650 fs transform-limited pulses (see bottom spectrum). The computed spectrum approximately spans from 4 µm to 11 µm at -10 dB of the peak PSD in the range of generated wavelengths. Such a broadband spectrum meets the need of various applications in the mid-IR including gas detection, molecular fingerprinting and hyperspectral imaging.

### Fiber laser source

**Fig. 2:**
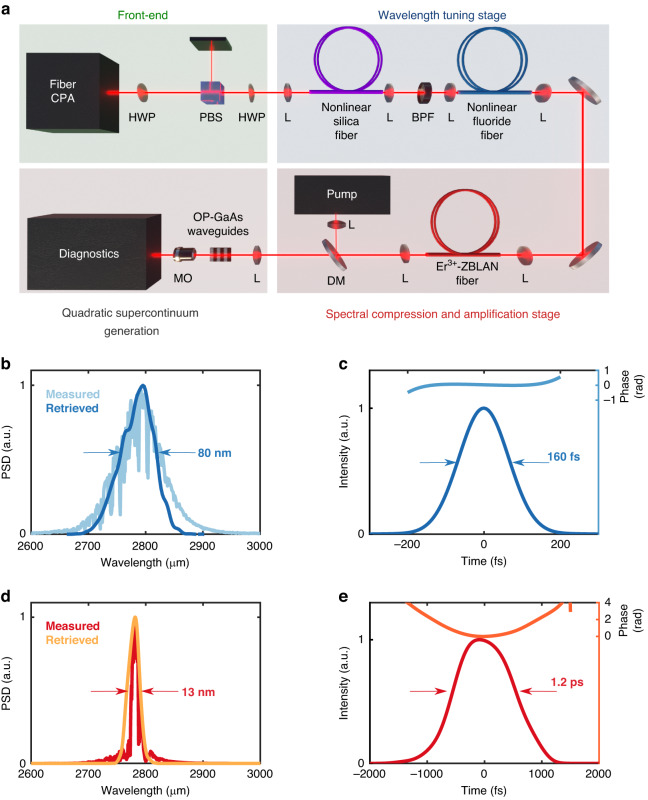
**Wavelength tunable picosecond fiber-based laser source. a** Schematic of the experimental layout used for mid-IR supercontinuum generation. A fiber-based laser source is first frequency-shifted up to ~2.75 µm and then spectrally compressed and amplified to match the optimal conditions for pumping an OP-GaAs-AlGaAs waveguide in the regime of group-velocity matching. HWP half-wave plate, PBS polarization beam-splitter, L Lens, BPF band-pass filter, DM dichroic mirror, WG waveguide, MO microscope objective. **b**–**e** spectral (left) and temporal (right) intensity profiles retrieved by FROG before (**b**, **c**) and after (**d**, **e**) the spectral compression stage. The temporal phases retrieved from the FROG traces are also shown on top of the temporal profiles

In this section, we detail our efforts to produce mid-IR high-energy transform-limited pulses in the picosecond range. The experimental setup is schematically depicted in Fig. 2a. A Thulium-doped fiber based front-end laser generates 765 fs pulses at 1965 nm wavelength and 1 MHz repetition rate. The radiation is launched into large mode area (LMA) fibers to produce an ultrashort pulse tunable in wavelength up to 3000 nm by multi-solitonic fission and soliton self-frequency shift (SSFS)^31,32^. The pulse was characterized by means of a home-built mid-IR frequency-resolved optical gating system based on second-harmonic generation (SHG-FROG). Independent measurement of the spectrum was carried out by means of a grating-based spectrometer (Yokogawa AQ6376) spanning from 1500 nm to 3400 nm and a Fourier-transform infrared (FTIR) spectrometer. After the SSFS stage, the pulse duration was measured to 160 fs at 3000 nm (see Fig. 2b-c). As shown in Fig. 1e, this ultrashort pulse is however not suited for efficient OPG in the waveguides due to the large group-velocity mismatch between the parametric waves and the pump wave (see Supplementary Material 1 for experimental results obtained when 160 fs pump pulses are launched into the fabricated waveguide). We have therefore developed a second stage in which spectral compression^33^ was used to increase the pulse duration. However, spectral compression occurs when a negatively chirped pulse propagates in a low nonlinearity medium. To achieve this situation in the fluoride fibers available for our study, we had to decrease the pulse energy by a factor of 20. In order to reach the threshold for broadband OPG requiring >10 kW peak power pulses, the spectral compression stage includes an in-house built Erbium-doped fluoride fiber amplifier (see Supplementary Material 2 for detailed results on spectral compression in mid-IR amplifier). By varying the pulse parameters in the SSFS stage (energy and central wavelength), we obtained several experimental configurations with a varying amount of spectral compression. The minimal spectral width and therefore the maximal duration of the pulse were 13 nm and 1200 fs, respectively (Fig. 2d, e). Pulse durations in the range of 600 - 800 fs were routinely achieved at a central wavelength of 2760 nm, with an energy per pulse in the range of 15 - 20 nJ. According to the numerical study, these performances are very close to the optimal operating point for broadband OPG.

### Supercontinuum generation

**Fig. 3:**
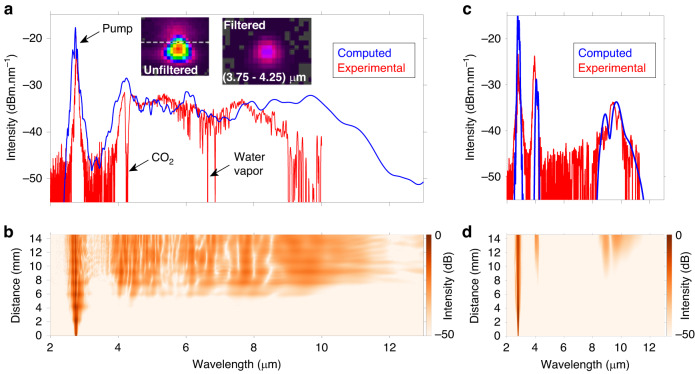
**Experimental demonstration of group-velocity matching mediated supercontinuum generation in OP-GaAs/AlGaAs waveguide. a** Supercontinuum generated in the 14.5 mm long OP-GaAs waveguide with *w* = 11 µm. The insets show the near-field intensity distributions recorded at the output of the waveguide without (left) and with (right) 500 nm band-pass filter centered at 4000 nm. **b** Computed evolution of the spectral profile along the length of the waveguide. **c** Conventional optical parametric generation spectrum obtained when the pump wavelength is detuned from the group-velocity matching regime (λ_P_ = 2790 nm, *w* = 12 µm). **d** Computed evolution of the spectral profile along the length of the waveguide

We have selected an experimental configuration with pulse duration around 650 fs because it represents a good trade-off between walk-off length (6.5 mm) and peak power (estimated to 12.6 kW). The 650 fs pulses were then launched into the OP-GaAs/AlGaAs waveguide with a rib width *w* of 11 µm and Λ = 85 µm. The spectral profiles at the output of the waveguide were measured by means of the FTIR spectrometer and plotted in Fig. 3. In this experimental configuration broadband OPG was recorded from 3900 nm up to 9300 nm, confirming that instantaneous broadband mid-IR generation can be achieved in QPM waveguides. The average power at the output of the waveguide was measured to 2 mW over the whole spectral range, including the residual pump. From the measured PSD, we estimate that the average power within the supercontinuum bandwidth amounts to 1 mW. In addition, the beam emitted by the waveguide is near-diffraction-limited, as shown in the insets in Fig. 3a. We have calculated that the spectral brightness of the source at the wavelength of peak PSD, i.e. 7700 nm, reaches 1.02 × 10^18^ ph.s^−1^.mm^−2^.sr^−1^.0.1%BW^−1^ (see “Materials and methods” for detailed calculation), surpassing that of third-generation synchrotron sources by a factor of 20 (5.04 × 10^16^) ph.s^−1^.mm^−2^.sr^−1^0.1%BW^−1^ (ref. ^34^). We then launched the pump radiation in a second waveguide with *w* = 12 µm. The spectrum is plotted in Fig. 3c. In this case, we observed conventional OPG with two distinct sidebands corresponding to signal (4 µm) and idler (centered at 9 µm). This behavior was already reported for our waveguides pumped beyond 3000 nm^25^. These observations confirm the fact that the waveguide and fiber laser must be designed in tandem to ensure group-velocity matching between the signal and idler waves around the degeneracy point and the subsequent supercontinuum generation in the mid-IR.

## Discussion

These results were also predicted by our numerical platform. We calculated the pulse energy coupled in the waveguide by considering the measured output power (2 mW), the transmission coefficient (0.72) at the GaAs/air output interface, and the transmission coefficient (0.356) along the 14.5 mm long waveguide assuming 3 dB.cm^−1^ losses. The estimated pulse energy coupled in the waveguide therefore amounts to 7.6 nJ, which corresponds to 12.6 kW peak power for 650 fs sech² pulses. Using these input parameters, we studied numerically the evolution of the pulse at 2760 nm into the waveguide with *w* = 11 µm and Λ = 85 µm and the results are shown in Fig. 3. The model is able to retrieve both the shape and the absolute PSD of the supercontinuum recorded in the experiments. However, we did not consider the free-space propagation of the beam towards the detector of the FTIR spectrometer, hence the absence of CO_2_ and water vapor absorption peaks in the computed spectrum. The dynamics of supercontinuum generation (see Fig. 3b) shows that in the group-velocity matching regime, the spectral range is generated as a whole after 7 mm of propagation. As shown in Fig. 3a, the theoretical bandwidth of the supercontinuum at -20 dB spans from 3700 nm to 10100 nm when 12 kW of peak power are launched into the waveguide. In our proof-of-concept experiment, the dynamic range of the measurement in the supercontinuum region, defined as the maximum value of the PSD divided by the detector noise floor, is however limited to 15 dB by the losses along the path to the detector of the FTIR spectrometer (estimated to 13 dB) and by the sensitivity of the detector, which could be improved with appropriate cooling, for example. On the other hand, a 30 % higher power could be launched into the waveguide using an anti-reflection coating on its input facet. Not only will this increase in peak power lead to an increase of the power density but also to a broader extension of the supercontinuum, as shown by our numerical simulations. We also expect that the higher peak power will trigger cubic nonlinearities. The interplay between quadratic and cubic nonlinearities, which is peculiar to OP-GaAs waveguides, might assist the spectral broadening towards the infrared. Moreover, if needed from the application side, the limitation of the PSD below the µW.nm^−1^ level can be circumvented by increasing the repetition rate of the source. For example, an increase of the repetition rate to 50 MHz, obtained from e.g. a commercial laser frequency comb at 1550 nm, which corresponds to a pump laser average power of 1 W, will increase the PSD to ~10 µW.nm^−1^. First of all, we have developed a continuous wave laser at 2750 nm and checked that the waveguides withstand 1 W power without any detrimental heating (the temperature of the input facet, recorded with a thermal camera, increases by 1 °C, which is not enough to modify the phase-matching curves). We have also demonstrated in a previous study that the seed used in the present study (2.5 nJ pulses at 2760 nm) can be delivered by an SSFS source based on a high repetition rate mode-locked source at 1550 nm^35^. The amplifier developed in the present work could therefore be used to both increase the duration of the transform-limited pulse and to amplify the energy up to the required level of ~20 nJ. We therefore believe that this approach is viable. Furthermore, the high coherence provided by parametric frequency down-conversion in quadratic nonlinear media makes this approach very appealing for frequency comb generation and few-cycle pulse production in the mid-IR.

## Conclusion

In conclusion, the main result of the present work is the demonstration of instantaneous broadband mid-IR generation in quasi-phase matched semiconductor waveguides pumped by a sub-picosecond fiber-based laser. Tailoring the propagation constant of an orientation-patterned GaAs waveguide in combination with the optimization of the output features of an ultrafast fluoride fiber-based pump laser enables broadband down-conversion close to the point of zero GVD of the QPM material. The spectral coverage which spans from 4 to 9 µm is well adapted to spectroscopy applications in the fingerprint region where many important organic (CO, CO_2_) and inorganic molecules (H_2_O, NO, O_3_, SO_2_) have their fingerprints. The brightness of the supercontinuum, which exceeds that of the third-generation synchrotron radiation source by a factor of 20, enables a range of applications, such as high-resolution micro-spectroscopy, that are today only accessible to large synchrotron facilities^34^.

## Materials and methods

### Fabrication of orientation-patterned GaAs/AlGaAs waveguides

First, an orientation-patterned GaAs template substrate is prepared using the wafer bonding technique. In this method^36^, the authors grow by MOVPE a stop layer and a thin GaAs layer (~100 nm) on a 2 inches diameter [001] wafer. This wafer is then bonded on another [001] wafer, i.e. with opposite crystal axis orientation. Next, the [00-1] side is etched down to the stop layer and this layer is removed so that only a thin [00-1] layer remains on the [001] substrate. Finally, the domain periods are defined by photolithography, and the patterned [00-1] layer is etched down to the [001] substrate to obtain the orientation-patterned template wafer. Then, in order to perform the regrowth of the AlGaAs/GaAs waveguide structure on this template, molecular beam epitaxy (MBE) is used, with growth conditions preserving the periodic crystalline orientation of the template. Starting with a 2.5 µm thick Al_0.5_Ga_0.5_As buffer layer, a 1.5 µm thick Al_0.3_Ga_0.3_As cladding is deposited before the guiding 14 µm thick OP-GaAs layer. The 7 µm deep orientation-patterned ribs are eventually defined using photolithography and inductively coupled plasma etching (BCl_3_ ICP-RIE). The final samples are cleaved from the obtained wafers and used in our experiments without anti-reflection coatings.

### Estimation of the brightness at 7.5 µm

From the power spectral density measured by the FTIR spectrometer and the independently determined total power of 1 mW above 4.5 µm we deduce a power of 1.54 × 10^–6^ W in 0.1% of the FWHM band around the wavelength of λ = 7.65 μm, which corresponds to a photon flux of 5.97 × 10^13^ ph s^−1^ 0.1%BW^−1^. Owing to the singlemodedness of the waveguide at 7.65 µm we assume that the mid-IR beam emerges from the waveguide with a radius of *w*_0_ = 6.5 μm, corresponding to an effective area of 120 µm^2^, a divergence angle of λ/(π*w*_0_) = 0.4 rad, and a solid angle of 0.48 sr. The resulting brightness is then 1.02 × 10^18^ ph.s^−1^.mm^−2^.sr^−1^.0.1%BW^−1^.

### Supplementary information

Supplementary Information for GaAs-chip-based mid-infrared supercontinuum generation

## Data Availability

All data needed to evaluate the conclusions in the paper are present in the paper and/or in the Supplementary Materials. Additional data related to this paper may be requested from the corresponding author.
