# Supplementary material for: GaAs-chip-based mid-infrared supercontinuum generation
Source: Light Sci Appl. 2023 Oct 18;12:252. doi: 10.1038/s41377-023-01299-9 (PMC10582246; doi:10.1038/s41377-023-01299-9)
Supplement: Supplementary file 1 — Supplementary Information for GaAs-chip-based mid-infrared supercontinuum generation [file 41377_2023_1299_MOESM1_ESM.docx]

**Supplementary Information for**

**GaAs-chip-based mid-infrared supercontinuum generation**

Geoffroy Granger^1^, Myriam Bailly^2^, Hugo Delahaye^1^, Cristian Jimenez^1^, Idris Tiliouine^1^, Yann Leventoux^1^, Jean-Christophe Orlianges^1^, Vincent Couderc^1^, Bruno Gérard^3^, Rezki Becheker^4^, Said Idlahcen^4^, Thomas Godin^4^, Ammar Hideur^4^, Arnaud Grisard^2^, Eric Lallier^2^, and Sébastien Février^1,*^

^1^ Université de Limoges XLIM UMR CNRS 7252, 123 Av. A. Thomas, 87060 Limoges, France

^2^ Thales Research & Technology, 1 Av. Augustin Fresnel, 91767 Palaiseau Cedex, France

^3^ III-V Lab, 1 Av. Augustin Fresnel, 91767 Palaiseau Cedex, France

^4^ CORIA (UMR 6614), CNRS-INSA Rouen-Université de Rouen Normandie, Normandie Université, Saint-Etienne du Rouvray, France

* corresponding author [sebastien.fevrier@unilim.fr](mailto:sebastien.fevrier@unilim.fr)

**1. Picosecond versus femtosecond excitation**

The efficiency of the parametric process depends on the group-velocity (v_g_) mismatch (GVM) between the parametric waves (located around the maximum v_g_ point, see Fig. 1c) and the pump wave. In our waveguide, the group-velocity mismatch between signal and idler pair and pump waves is $\mathrm{GVM}=\frac{1}{\frac{1}{v_{g}^{s,i}} - \frac{1}{v_{g}^{p}}}=$ 100 fs.mm^‑1^. The temporal walk-off induced by GVM in our 14.5 mm long waveguide therefore strongly affects the interaction when we use short pulses. For example, we have plotted in Fig. S1, blue, the spectrum when 160 fs are launched into the waveguide with *w* = 11 µm. The blue curve shows no evidence of OPG (the peaks at 1962 nm and 2370 nm are related to the unfiltered residual pump (see Fig. 2a, front-end laser and first stage SSFS). It is clear from Fig. S1 that the 160 fs pulse excitation manly leads to self-phase modulation around the pump wavelength, which is confirmed by the numerical study (see Fig. 1e, spectrum at the top).


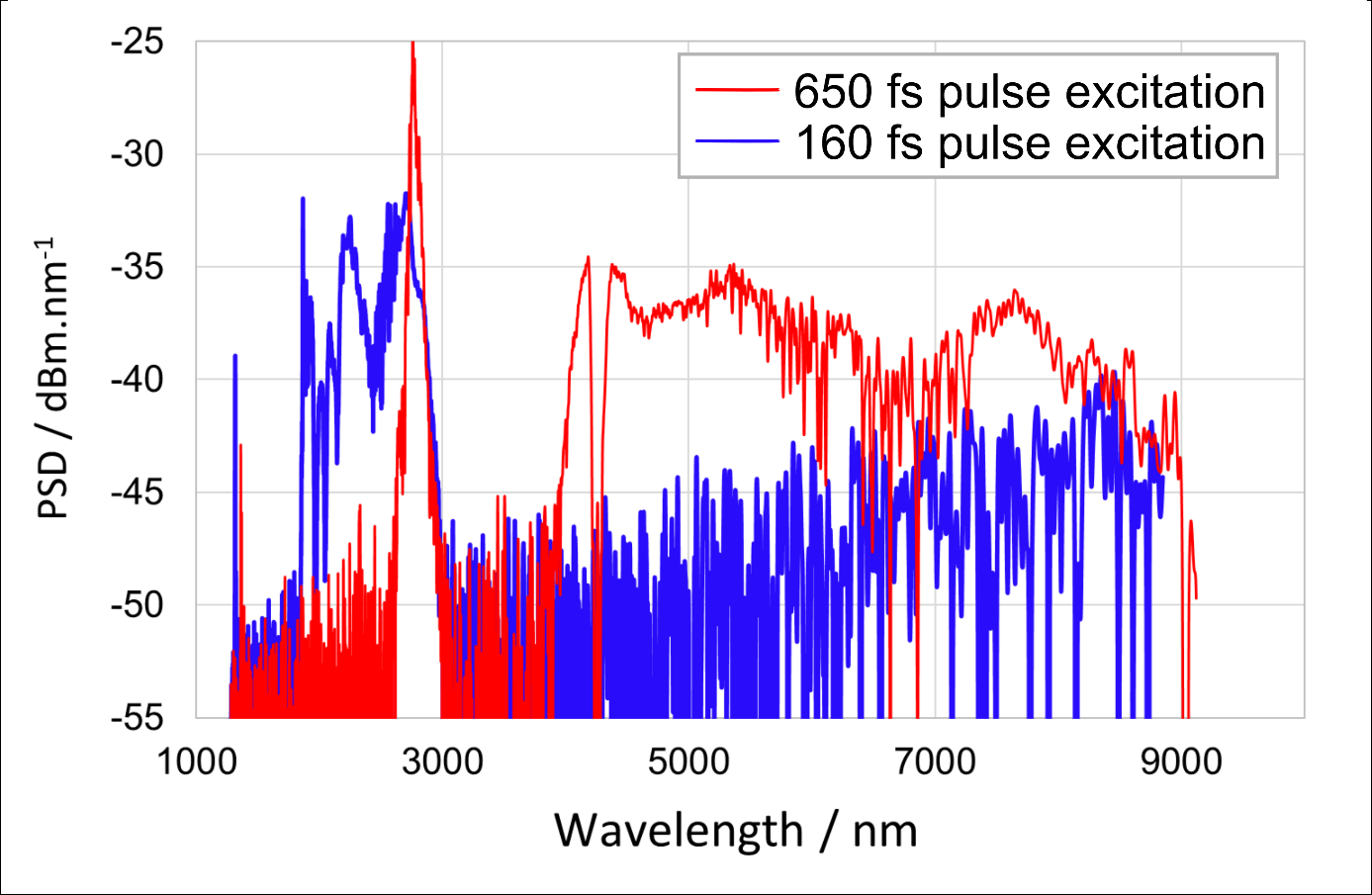


**Fig. S1** Comparison between the spectra recorded at the output of the 14.5 mm long OP-GaAs waveguide with *w* = 11 µm for two different excitation sources. In blue: before the compression stage (pulse duration is 160 fs, see Fig. 2b-c) and in red, after the compression stage (same spectrum as in Fig. 3a).

**2. Spectral compression in the mid‑IR**

The strong temporal walk-off induced by the group-velocity mismatch can be mitigated in short waveguides (length < 2 mm), but at the cost of an increase of the input peak power necessary to overcome the low conversion efficiency. The increase in input pulse peak power is however limited by the onset of laser-induced damage at the waveguide facet. Increasing the duration of the transform-limited pump pulse to the picosecond range, on the other hand, relaxes constraints in terms of group-velocity mismatch and enables the use of centimeter long waveguides pumped with reasonable peak power pulses. Nonlinear spectral compression provides a means to transform broadband femtosecond pulses into near-transform-limited picosecond pulses. Spectral compression results from the propagation of a negatively chirped pulse into a nonlinear Kerr medium, where the self-phase modulation (SPM) redistributes the high and low frequency components toward the center of the spectrum^1^. Importantly, the near-transform limited feature of the pulse is preserved upon SPM-induced spectral compression, with positive implication in the case of OPG in centimeter long waveguides. This effect was observed at low energy level in passive fibers^1,2^. High-energy transform-limited pulses were also produced by nonlinear spectral compression in Ytterbium-doped fibers at 1 µm (Ref. 3). Here, we exploit spectral compression in the mid-IR for the first time to our knowledge.

**Modeling results**

We have studied several configurations leading to the generation of pulses in the picosecond range at 2.75-2.76 µm. Spectral compression occurs in an anomalous dispersion fiber operated in the low nonlinearity regime, characterized by a small solitonic number slightly below unity. In our study, this configuration is obtained at the cost of a drastic reduction of the energy of the pulse at the output of the SSFS stage. In the numerical study, we therefore considered 160 fs pulses with 2.5 nJ energy (20× attenuation). We started the study with a passive stretching fiber located after the Raman frequency-shifting fluoride fiber. The fiber was a 1.6 m long single-mode fluoride fiber with 14 µm core diameter and 0.12 numerical aperture (NA). The pulse propagation was characterized by a nonlinear length L_NL_ = 20 cm, a dispersion length L_D_ = 9 cm, and a soliton number $N=\sqrt{{L_{D}}/{L_{\mathrm{NL}}}}=0.67$, confirming that the fiber is operated in the low nonlinearity regime. The results of the numerical study are shown in Fig. S2. As shown in Fig. S2c-e, the input 160 fs pulse was stretched to about 3 ps. A small amount of SPM-induced spectral compression can be seen in Fig. S2a-b. Nevertheless, the passive fiber mainly acts as a stretcher for the experimental parameters. As shown in Fig. S2f, the time-bandwidth product of the pulse is as high as 6, confirming that the propagation is mainly dispersive and that this first configuration does not lead to the formation of picosecond pulses with high power spectral density. We have studied longer fibers and higher input energy, without striking improvement. We have then decided to include an Erbium-doped ZBLAN fiber amplifier, as studied previously at 1 µm^3^. As shown in Fig. S2a-e, strong spectral compression occurs in the amplifier and leads to the formation of 8.7 nm bandwidth pulses with 1.24 ps duration. In this case, the time-bandwidth product decreases to 0.6, indicating that the pulse is near-transform-limited. The pulse energy increases to 14 nJ, corresponding to a peak power of 10 kW. According to the results of the numerical study shown in the main text (Fig. 1e), the power spectral density of the pulse is compatible with supercontinuum generation in the fabricated waveguide.


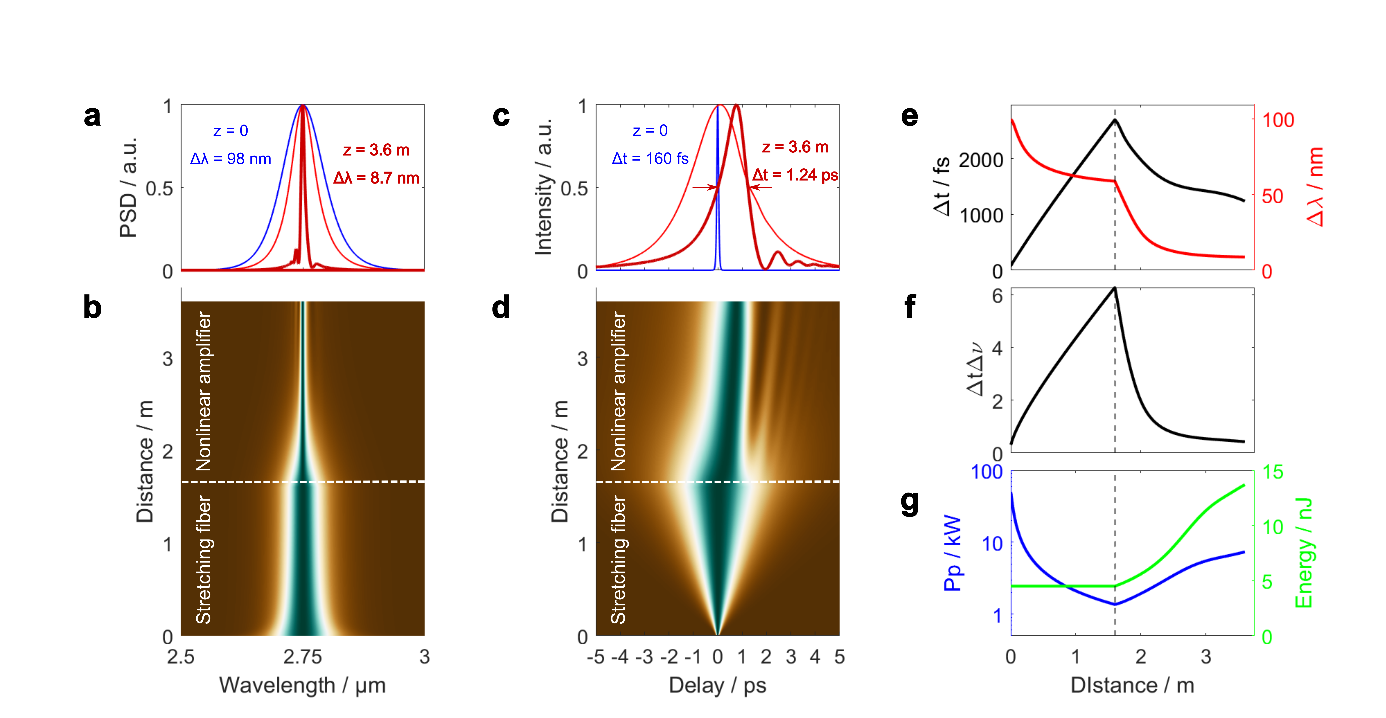


**Fig. S2** Modeling results of spectral compression around 2.75 µm in a two-stage architecture.

Nevertheless, a simpler architecture was studied, in which the passive fiber was removed. In this case, the input pulse is strongly negatively chirped in the first 40 cm of the active fiber (Fig. S3d-f). The large temporal broadening of the pulse associated with a level of population inversion in the backward pumped amplifier, leads to a low nonlinearity propagation medium, favoring spectral compression in the first part of the amplifier (approx. 1m). Then, the pulse energy is amplified almost linearly with the propagation length up to 20 nJ (Fig. S3g). The pulse duration at the output of the amplifier is 920 fs. The time bandwidth product of the pulse is kept low (0.6) showing that the pulse is also close to transform-limited. The final pulse duration is slightly shorter than that obtained when the passive stretching fiber was included. Nevertheless, the estimated peak power of approx. 15 kW is higher and commensurate with supercontinuum generation in our OP-GaAs waveguide. This simple architecture was chosen for the experiments.


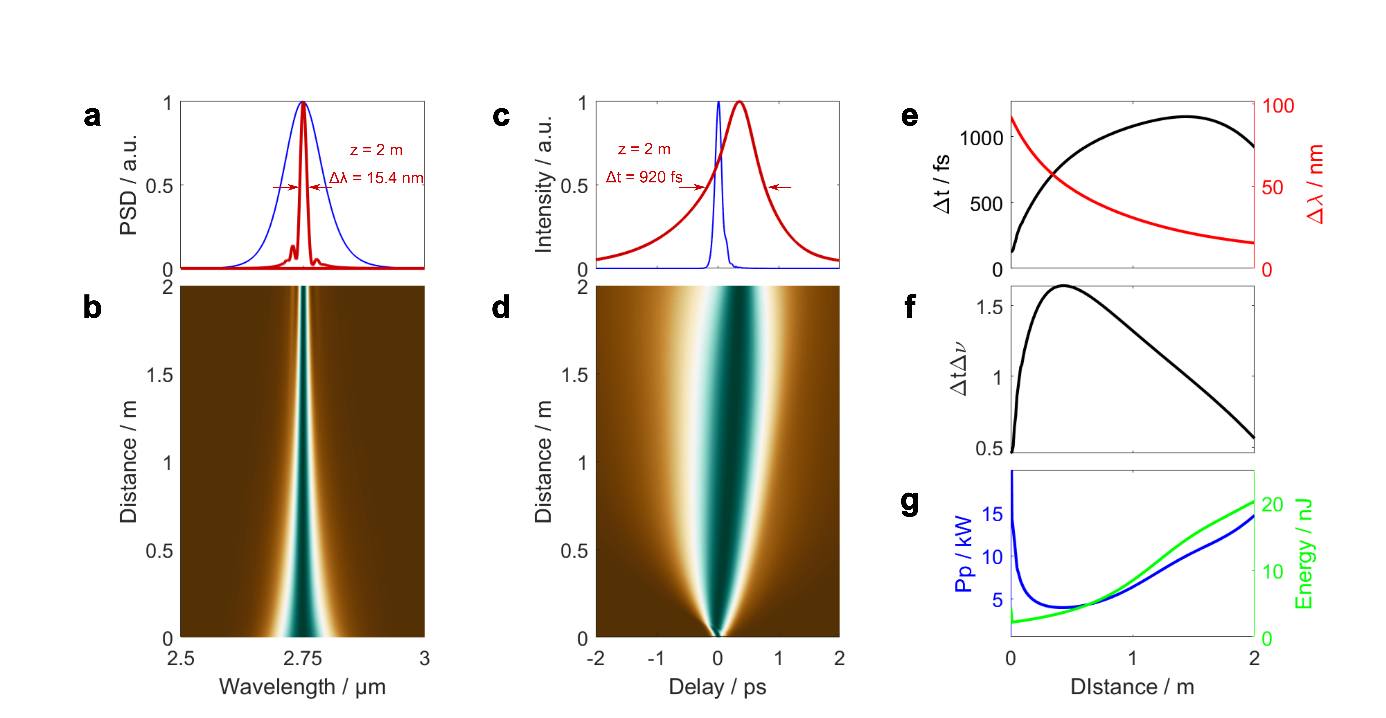


**Fig. S3** Modeling results of spectral compression at 2.75 µm in a single active fiber.

**Experimental results**

According to the numerical study, we have developed an in-house built 2 m long nonlinear Er-doped fluoride fiber amplifier. The pulse exiting the Raman frequency-shifting fluoride fiber was attenuated to 2.5 nJ and launched into a 15 µm core diameter fluoride fiber, doped in erbium with a concentration of 7×10^4^ ppm (*Le Verre Fluoré*, France). The fiber segment was coated with a low-index fluoroacrylate polymer, which allows pump guiding with a NA of 0.46. The amplifier was counter-propagation cladding-pumped by 980 nm pigtailed multimode diodes launched into the 260 µm cladding with double-D shape for optimal pump mixing. We found experimentally that the quality of the spectral compression was maximized by properly adjusting the level of backward pump sent into the amplifier to 2 W, corresponding to an overall signal gain of 9. For too high an optical gain, a compensation of the negative chirp by self-phase modulation occurred for shorter fiber lengths, leading to spectral broadening and even to multi-solitonic fission towards the end of the amplifier. We have measured the temporal and spectral profiles of the pulse for various levels of backward pump power. A selection of results are displayed in Fig. S4. The pulse can be temporally broadened to 1200 fs for a 13 nm spectral width. These results are in good quantitative agreement with the numerical predictions shown above.

**
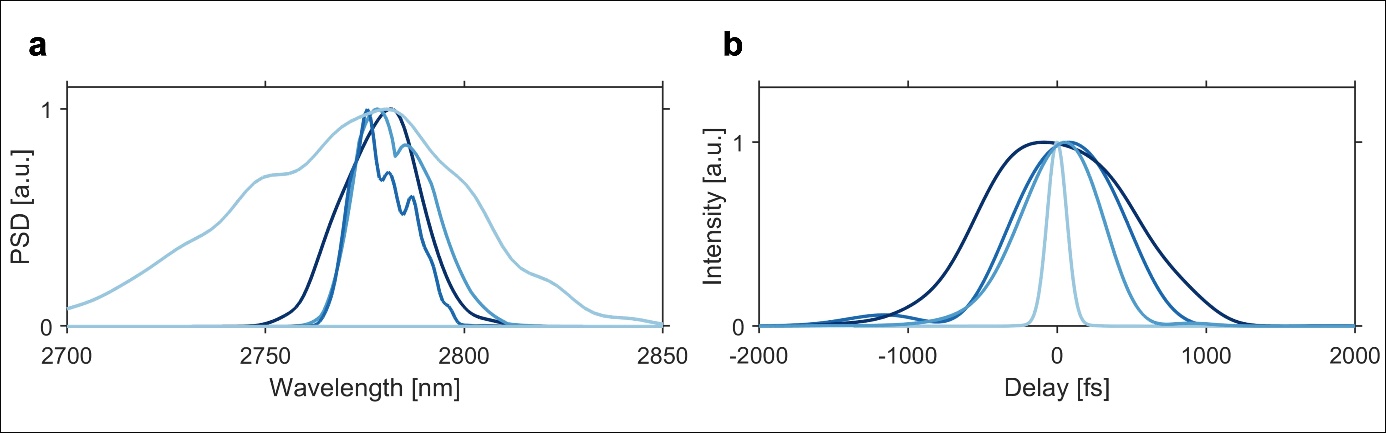
Fig. S4** Spectral (a) and temporal (b) profiles of the pulse at the output of the compression stage for various experimental configurations.

**References**

1. Oberthaler, M. & Höpfel, R. A. Special narrowing of ultrashort laser pulses by self-phase modulation in optical fibers. *Applied Physics Letters* **63**, 1017-1019 (1993).

2. Washburn, B. R. *et al*. Transform-limited spectral compression due to self-phase modulation in fibers. *Optics Letters* **25**, 445-447 (2000).

3. Limpert, J. *et al*. SPM-induced spectral compression of picosecond pulses in a single-mode Yb-doped fiber amplifier. *Applied Physics B* **74**, 191-195 (2002)
